# Fallacy of the Change of Type Theory

**Published:** 1864-04

**Authors:** 


### FALLACY OF THE CHANGE OF TYPE THEORY.

Opposed, however, to these ideas is a doctrine which has recently been put forth by a late distinguished Edinburgh professor (Alison,) and supported to a large extent by the senior members of the profession. This doctrine is, not that recent changes in practice result from an improved knowledge, or an advance in diagnosis and pathology, but that diseases themselves change. He thought, for example, that inflammation is no longer the same now that it was in the time of Cullen and Gregory; that the human constitution (in a manner which he did not explain) is fundamentally altered, and has become weaker; so that medical men were as right in treating disease by blood-letting in former days as they are now in abstaining from it. So satisfactory does this theory appear to its supporters that they have claimed for it the dignity of an ultimate fact or axiom. Thus, says Dr. Alison, changes of type in inflammatory diseases constitute "a part of the general dispensations of Providence as to those diseases, and are, as far as yet known, *an ultimate fact in their history.*" Dr. Watson says, no less emphatically, in the last edition of his work on the "Practice of Physic": "I am firmly persuaded, by my own observations and by the records of medicine, that there are waves of time through which the sthenic and the asthenic characters of disease prevail in succession; and that we are at present living amid one of its adynamic phases."

Let us for a moment consider what this theory implies, viz: that the constitution of mankind has become weaker and less capable of bearing depletion now than formerly; that the human pulse, by which this is tested, beats less vigorously when diseased than it did for hundreds of years before the days of Cullen and Gregory; that when a strong man now-a-days is seized with an inflammation he presents all the phenomena that used to be observed in a weak one; in short, that the human race has so degenerated during the last five-and-twenty years, that the re-action which formerly used to take place in the economy no longer occurs, and that it cannot bear depletion so well.

But surely this idea may be said to repose on no facts whatever, but merely on supposition; for when we investigate the effects of injuries after the battle of Waterloo and after the battle of the Alma, we find them in the British army identically the same. Neither has any change been observed in this respect in our city hospitals. Then the people generally are better fed, clothed, and housed than they used to be; the comforts and enjoyments of life are far more widely diffused, and its absolute value, according to the bills of mortality, is greatly augmented. Our mental strength, commercial enterprise, engineering skill, martial daring, and bodily vigor might easily be shown never to have surpassed what this country can now boast of—facts entirely opposed to this theory.

Then the treatment of inflammation without anti-phlogistics has also changed for the better among veterinary practitioners. Is it to be maintained, therefore, that our horses and cattle have also, as the result of civilization, been innervated, and that in them also the type of disease is altered? We nowhere observe this any more among them than among mankind; they still draw the same loads—still plow with the same depth of furrow—still run with the same if not greater speed.

Besides, it should not be forgotten that the anti-phlogistic has been shown to be a fatal practice—in acute pneumonia amounting to one death in three cases. Since the practice has been changed, the deaths only amount to one in twenty or thirty, as I have previously shown. To prove that this is a result of treatment. and not of change of type, it is only necessary to consider that in countries such as Spain and Italy, where medical science has not advanced in the same ratio as it has done with us, the old practice is still followed, and with the same fatal result. Have we not all recently been startled by the death of Count Cavour, which followed five bleedings for a fever? Are we then to believe that, whilst the people of Britain, France and Germany, have degenerated, those of Spain and Italy have retained their pristine vigor? In Paris, M. Bouilland continues to pursue his system of bleeding by the *coup-sur-coup* method. He is the only one in that capital who does so. Can we on this account believe that in his wards the type of disease has not changed, whilst in every other hospital and ward it has? On the contrary, we find that wherever large bleedings are practiced at present, the same great mortality prevails as used to prevail, showing that the disease is still the same.

Then it has been argued that epidemic fevers change their type, and so they unquestionably do, but it in no way follows that organic diseases should do so likewise. The morbid poisons in tne atmosphere arising from various sources are more powerful at one period than another, and not only induce symptoms varying in intensity, but cause varied symptoms, such as occur in typhus or typhoid fevers. It is the latter changes which constitute difference in type. But there have been strong and weak men in all ages; while blows, injuries, and changes of temperature have similarly affected them, occasioning symptoms proportioned to their bodily vigor, but in no way altering the character of the symptoms themselves. Have cancer, tubercle, or other structural changes undergone a change of type?—or is it necessary to explain the effects of an improved practice, to assert that, while persons affected with inflammations are now weaker, those affected with phthisis and scrofula are stronger than they used to be.

But it is stated that the pulse has altered; formerly it was found to be strong, now it is comparatively weak. Why, within the last twenty-five years, nature should have changed the pulse of man and animals is not very clear. Judging from the circumstances to which I have alluded, especially the more abundant food and prosperity of the people, it ought to be stronger instead of weaker. But some have already brought forward ideas to explain the supposititious fact. Thus it has been said the use of tea instead of malt liquor, spirits, and wine, renders people weaker and more nervous. Some have thought that the use of potatoes, and others the employment of railways, has something to do with it. Dr. Watson is of opinion that it is attributable to the epidemics of cholera, which, in a manner he has not sought to explain, "leave traces of their operation on the health and vitality of a community long after they have ceased to prevail as epidemics." (Pneumonia, vol. ii, p. 97.) Mr. Robertson of Manchester, is satisfied from experience that it is the boil epidemic which has caused this remarkable change of type. Some suppose that it is dependent on the altered relations between our urban and rural population.—Would it not be well for those who are already discussing the causes of a change that is by no means apparent, to ask themselves, in the first instance, how they establish the fact that the pulse is changed at all?

I need scarcely say that memory and mere opinion in a case of this kind are not of much value. How often do our senses deceive us when objects are at hand; how little can they be depended on whan it is simply asserted, that in the memory of this or that practitioner a pulse was stronger twenty years ago than it is now. And yet, gentlemen, we have no further evidence than this advanced by the supporters of a theory which claims as its fundamental fact a diminished vital force in the heart and pulse of man and animals, to explain a change of practice. But what say science and positive observation to these assertions? It so happens that there is no subject in all physiology with regard to which we possess more elaborate and more exact information than we do concerning the pulse. One hundred and twenty years ago Hales published a remarkable series of experiments regarding the static force of the pulse, and the rapidity of the blood through arteries of different calibres; and similar observations were made by Poisseulle with an instrument invented for that purpose, which he called the "hæmadynamometer," that led him to the same conclusion as that arrived at by Hales. In these experiments the force of the pulse was determined by the height which the impulse of the blood could elevate a column of mercury. It resulted that the static force with which the blood is impelled in the human aorta is equal to the pressure of 4 lb. 4 oz. on the square inch, and in the radial pulse is equal to about 4 drachms.—Valentin confirmed these results in 1844, Ludwig in 1847, and Vierordt so late as 1855; so that not only is there no fact whatever in support of the notion that the pulse of man or animals is weaker now than formerly, but all positive researches during a period of one hundred and twenty years prove the very contrary. It appears to me, therefore, that the theory of change of type, so far from being established on well-known facts, is, on the contrary, altogether fallacious, and entirely opposed to all the known data which histology, physiology and pathology, have accumulated in modern times.—*London Lancet.*

